# Experience with an online helpdesk for equipment fault reporting in a radiation oncology department

**DOI:** 10.1120/jacmp.v14i6.4396

**Published:** 2013-11-04

**Authors:** Joanne McNamara, Matthew Williams, Martin Carolan

**Affiliations:** ^1^ Shoalhaven Cancer Care Centre Nowra NSW Australia; ^2^ Illawarra Cancer Care Centre Wollongong NSW Australia

**Keywords:** radiotherapy, help desk, safety, fault

## Abstract

The safe and accurate delivery of radiotherapy requires that the various items of equipment used in the treatment process are in proper working order. Unusual equipment behavior, interlocks, and faults need to be communicated promptly to the appropriate personnel, the issue investigated, and information regarding the nature of faults and any subsequent reparative measures recorded in a readily accessible location. At the Illawarra Cancer Care Centre, an online help desk was introduced for the purposes of reporting and tracking equipment faults. In the three years since its introduction, there have been in excess of 1,300 entries made, and it has proven to be a suitable alternative to the use of a physical logbook. Key benefits identified were improved accessibility, automated fault notification, and the ability to search, review and update entries for particular equipment faults. It has been a useful tool for assessing the long‐term performance of each piece of equipment, identifying specific servicing needs, and for substantiating the effectiveness of the service performed. The online help desk has not replaced the need for direct methods of verbal communication between the various professional groups involved with monitoring equipment performance. However, it is a very useful tool for supporting that communication.

PACS number: 87.55.‐x, 87.56.bd

## I. INTRODUCTION

In a radiation oncology department, a variety of complex equipment is used during the course of a patient's treatment. Over time, various components of the equipment can degrade, become damaged, or fail. There can also be direct or indirect changes to the operational environment that can have an impact on the performance of the equipment. To ensure reliable and safe functioning of the equipment, there should be routine preventative maintenance performed, and adherence to appropriate commissioning and quality assurance recommendations.^(1)^ Another key component of ensuring reliable performance of equipment is to have informed and efficient responses to errors, faults, and interlocks. Having a clear communication strategy for the reporting and investigation of equipment faults has been identified as a crucial step in the prevention of accidental radiation exposures,^(^ ^2^ ^,^ ^3^ ^)^ and it is a regulatory requirement in Australia that a written record is kept detailing the work performed on radiation‐producing equipment following any repair, maintenance or modification on that equipment.^(4)^

It had been standard practice at the Illawarra Cancer Care Centre (ICCC) for all items of radiation apparatus to have a logbook. In the event of a fault, interlock or other unusual occurrence, staff members were required to notify the medical physics department and document the details of the event in the logbook. Similarly, the details of any modifications or repairs made to the equipment were also to be documented in the logbook. A limitation to this approach was that the logbook was often considered to be a secondary form of communication, and although all major events were orally reported, not all events were recorded. Conversely, if the event was only minor and treatment was uninterrupted, then it was not unusual for an entry to be made in the logbook and the medical physics department notified at a later time.

The effectiveness of the a book in capturing and communicating information regarding equipment performance is dependent on an entry being made in the logbook, the quality of the data recorded, and staff routinely reviewing the logbook. There was a need for an easily‐accessible system with set data requirements to ensure that sufficient information was provided and each fault could be identified quickly and linked to appropriate personnel. An electronic system with notification and analysis capabilities was thought to be ideal for this purpose. As with other quality management methods such as six sigma and lean thinking, which are similarly used in a health‐care setting to improve quality and refine process,^(^ ^5^ ^,^ ^6^ ^)^ the ability to quickly view, trend, and analyze data is critical.

In June 2009 an online help desk software program, known as SysAid (SysAid Technologies Ltd., Or‐Yehuda, Israel), was implemented at the ICCC. The intention was that the online help desk software would fulfill the role of the logbook and provide a means for recording, monitoring, and managing equipment faults. This paper provides a basic overview of the software functionality, describes the ICCC experience using it for the reporting of equipment faults, and provides some examples of how SysAid was used in the management of specific equipment issues.

## II. MATERIALS AND METHODS

The SysAid software is promoted as a help desk solution for information technology (IT) departments. The system provides tools for the management of computing hardware and software, and provides service desk functionality.^(7)^ It is a Web‐based application with its own web server, and clients access the help desk via an Internet browser. Although the software is designed primarily for an IT environment, there are several key elements that made it attractive as a fault reporting system for radiotherapy. Of particular interest when evaluating SysAid for radiotherapy purposes were the user and group management tools, the simplicity of the user interface, the level of customization available, its fault management capabilities, and data analysis tools.

### A. User management

In a radiotherapy department, the primary users of radiation apparatus are radiation therapists, physicists, and engineers. These front‐end users are the groups most likely to encounter equipment faults. In regard to the investigation and rectification of faults, it will be the local physicists and engineers who are responsible. To manage and follow up faults adequately, it is important that the individuals involved in a fault are clearly identified. In our implementation of SysAid, each individual has been set up with their own username and password. The software allows for several different levels of users, each with an increasing level of security and access rights. Most users, such as radiation therapists, have been designated as end users. These users have limited access rights and can make new entries (referred to as service requests) and change their personal settings. Senior radiation therapists are designated as supervisors and can view the current status of all open service requests within their area of responsibility. Physicists and radiotherapy managers have been assigned as administrators. Administrators are responsible for the management of service requests, and have a variety of higher level permissions relating to the ability to view and modify service requests and generate reports.

In SysAid, users are assigned within a hierarchical group structure to entities referred to as “companies”, “departments”, and “groups”. Companies offer a way to segregate particular parts of the help desk. If a user belongs to a company (or companies), the categories of equipment visible to them within the help desk software is controlled; the specification of these categories is described in the following section. Each company has its own set of administrators. An example of two companies defined in SysAid for a radiation therapy center may be the radiotherapy treatment machines and the oncology information system (OIS). To further illustrate this example, a radiotherapy nurse who does not require access to the treatment machines could be assigned to the OIS company; therefore, when they log an issue using SysAid they are only presented with options related to the OIS. The OIS problem would then be assigned to the administrators responsible for that “company”. A radiation therapist uses both the treatment machines and OIS and, therefore, would belong to both companies and have access to options for both. The access of external service providers to the system could also be managed using this functionality; however, in our implementation access has been restricted to radiotherapy and biomedical personnel.

### B. Customization

**Figure 1:**
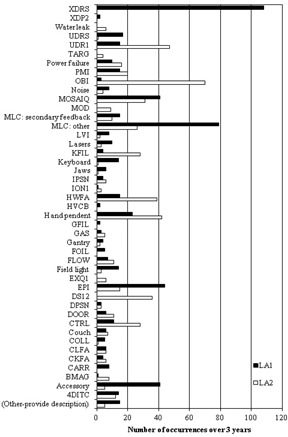
The distribution of machine interlocks occurring on two different linear accelerators at the Illawarra Cancer Care Centre over a three‐year period. For a detailed explanation of each acronym and fault, refer to the Varian User Manual.^(8)^ 362

For the system to be useful in the radiotherapy setting it had to provide comparable or improved convenience over the use of a logbook; it had to be simple, intuitive, and capable of handling radiotherapy specific jargon and nomenclature. The SysAid end‐user portal presents fault reporting options through a series of dropdown menus. For our purposes, each radiation device has been defined as a category and, within each of these, further subcategories have been defined that are associated with a specific machine fault. The machine fault dropdown list is comprised of the known errors that have been documented by the manufacturer, as well as other faults and events that may conceivably be encountered on the machine. A sample of the subcategories available for the linear accelerator is shown in Fig. 1. The categories and subcategories are highly customizable, and the administrators have added extra categories as the need arose. When creating a service request, there is also a section for the end user to add a free‐text description and the option to attach files such as log files, images, screen‐captures, and documents. The use of categories and subcategories allows for particular faults to be easily identified and followed up by administrators, and the free text allows for more detailed situation specific information to be recorded.

### C. Fault notification chain

**Figure 2:**
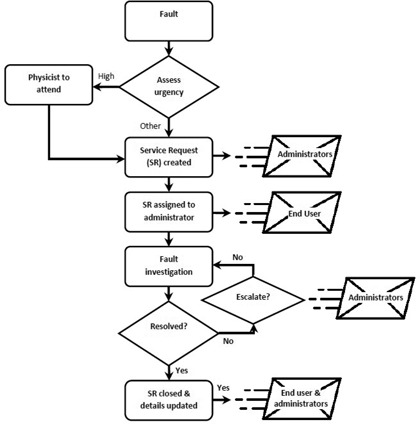
The fault notification chain that is implemented at the ICCC. The system has been configured to provide automated email notification when certain conditions are met.

In Fig. 2, a schematic of the fault notification chain used at the ICCC is shown. When a fault occurs, the appropriate staff members need to be notified as soon as possible. For the linear accelerators, if a major dosimetry interlock occurs or the machine is not operational, then a physicist is required to attend; at the same time a service request is entered in SysAid. Upon creation of a service request, the user must specify the urgency; this particular type of linear accelerator fault is assigned the highest urgency. For all other faults, an entry is made in SysAid and an appropriate urgency assigned as determined by the user. Following the creation of a service request, the administrators can be automatically notified via email or SMS.

As part of the SysAid configuration, a set of routing and escalation rules, due dates, and alerts are defined for each category and the subcategories. The configuration of these rules allows for the administrators, and end users if required, to be automatically assigned or notified when certain conditions are met. For instance, an escalation alert can be issued to the administrators if the status of a service request has not been updated within the designated time frame. Similarly, when action is taken, such as a note being added, a particular person assigned responsibility, or extra files attached to the entry, an email can be sent. At the ICCC, once responsibility is assumed for a job, the service request is manually assigned in SysAid. All administrators, regardless of their designated responsibility, are notified by email when a fault is created or updated; hence, all administrators are made aware of machine problems as they occur and any measures taken to fix the problem. The system was deliberately set up this way as it is particularly valuable when fault rectification takes place over an extended period of time and helps facilitate the handover of information between staff involved in managing the fault. The decision‐making process cannot be eliminated from this system; it relies on the professionalism of staff to recognize and report the fault, and to assess and decide on the appropriate urgency. The physicist must then ascertain if reparative measures are necessary and decide on actions to take to rectify the problem.

### D. Database features

The system can be configured to use an in‐built database or interface with an external database such as Oracle Database (Oracle Corporation, Redwood Shores, CA) or Microsoft SQL Server (Microsoft Corp., Redmond, WA). An internal database option was chosen at ICCC, accessible only by members of the radiation oncology department. This alleviates patient privacy concerns by ensuring that only members of the department have access to any patient‐related information recorded with a fault. This is useful for quickly identifying problems that may need attention on planned maintenance days, as well as diagnosing infrequent but persistent problems. There is a knowledgebase section of the database where solutions for commonly encountered problems can be detailed; the knowledgebase can be readily searched by end users (if permitted) and administrators to quickly rectify a fault. There is also a variety of predefined and custom reports that can be utilized for analyzing data.

## III. RESULTS

There have been over 1,300 service request entries in the ICCC system since it replaced the paper logbook. The data shown in the following figures have been generated using the in‐built reporting functions of the SysAid software. A comparison of the faults recorded for the two linear accelerators at the ICCC over a three‐year period from June 2009 to July 2012 is shown in Fig. 1. The entire list of subcategories is not shown and only faults that have occurred at least once during the three‐year period are included. The majority of acronyms listed refer to specific Varian linear accelerator faults; explanation of these faults is provided in the user manual.^(8)^ The linear accelerator ‘LA1’ is a Varian 21EX machine equipped with multileaf collimators (MLCs) and an electronic portal imager (EPI) (Varian Medical Systems, Palo Alto, CA). In March 2012 this linac was retrofitted with a kilovoltage imaging device known as an on‐board imager (OBI). The linear accelerator ‘LA2’ is a Varian CL‐iX machine equipped with MLC and OBI. The comparison between the machines reveals that LA1 has had considerably more excess dose rate (XDRS), MLC and EPI faults than LA2 and LA2 has had significantly more under dose rate (UDR1), OBI and dosimetry (DS12) interlocks.

### A. Differences in machine usage

The disparity in MLC interlocks between the two machines reflects that LA2, with the more recent MLC and later software version, had more reliable performance. The disparity of a high number of electronic portal imager (EPI) faults on LA1 and a high number of OBI faults on LA2 is a reflection of the difference in image acquisition between the two linacs. The OBI was not installed on LA1 until March 2012 and, prior to that, the EPI had been the primary and only imaging device, while on LA2 the OBI had always been the primary imaging device.

### B. Effectiveness of service

**Figure 3:**
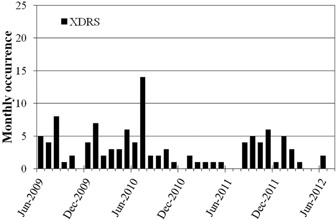
Monthly frequency distribution of excessive dose rate (XDRS) interlocks occurring on linear accelerator ‘LA1'

A detailed histogram of the LA1 XDRS interlocks for the three year period is shown in Fig. 3. An XDRS interlock indicates that the internally measured dose rate over a given period of time exceeds the intended rate.^(6)^ An analysis of the SysAid records revealed that at the peak of the occurrences in July 2010, the corrective measure of fine tuning the radiofrequency (RF) of each photon energy was performed. The tuning of the RF did result in an immediate decrease in the number of XDRS interlocks; however, the interlock continued to occur one to four times a month. It is not until the interlock is chronologically reviewed that it becomes apparent that there is an underlying problem that was not addressed by the tuning. The ability to quickly generate this type of information is very useful for ensuring that faults are investigated correctly, but it also is a valuable tool for evaluating the effectiveness of servicing. Service support contracts provided by linear accelerator vendors can be a significant operating expense for a radiotherapy center. The management and performance evaluation of the vendor service support is enhanced by having access to detailed data on the frequency of faults and the time taken to resolve them.

**Figure 4:**
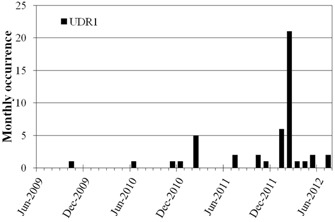
Monthly frequency distribution of UDR1 interlocks on linear accelerator ‘LA2', which relates to the radial dose rate being less than expected.

The field service reports provided by the engineers are scanned in by local personnel and can provide missing pieces to the puzzle when trying to identify the cause of machine problems. The occurrence of UDR1 interlocks on LA1 is shown in Fig. 4, and there is a noticeable spike occurring in February 2012. A UDR1 interlock occurs on a Varian linac when the radial dose rate is less than 80% of the set value.^(8)^ A review of the SysAid records indicates that in January 2012 during routine preventative maintenance, it was observed that a capacitor in the pulse forming network of the accelerator was physically bulging. It was following the replacement of this capacitor that the UDR1 interlocks started. According to the service records contained in SysAid, the subsequent investigative work performed by the vendor on the UDR1 interlock attributed the fault to the replacement of this capacitor and the incomplete testing of the pulse forming network following its replacement.

### C. Faulty components

**Figure 5:**
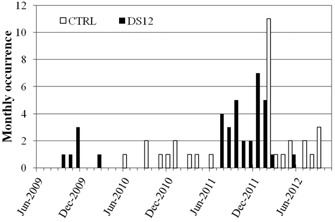
Monthly frequency distribution of a dosimetry interlock (DS12) and control computer (CTRL) interlock on ‘LA2'.

By tracking specific faults, the relationship between a particular fault and machine components can be better understood. A DS12 interlock is a dosimetry interlock that indicates that the internal dose monitoring system has detected a discrepancy in dose or has failed. This particular interlock is considered a major interlock of the highest urgency that requires direct involvement of a physicist. On LA2 the DS12 interlock most recently started to occur in July 2011; however, it had occurred for a period from 2009 to 2010. These periods coincided with the replacement of a printed circuit board (PCB) that was part of the timing circuitry used to control the dosimetry system. Upon discussion with the vendor, it was discovered that the DS12 interlocks were related to a newer version of the PCB, however the older version of the PCB was causing control computer (CTRL) interlocks. There was some concern that treatment staff might become complacent with recurring dosimetry interlocks. Therefore, in February 2012, the new version of the control timer PCB was replaced with the older model of the board, reducing the DS12 interlock frequency but reintroducing CTRL interlocks. The relationship between the two faults and the correlation with the PCB component has been recorded in SysAid and is shown in Fig. 5. Accurate records of ongoing fault related interlocks can assist local engineers and physicists in providing feedback to the vendor regarding outcomes of service interventions.

## IV. DISCUSSION

The use of an online help desk system has proven advantageous over the use of a conventional logbook. The capacity to quickly review and search for specific faults has allowed for long‐term trends to be more readily discovered and correlation with QA results made. The main features that have been of benefit are the accessibility of the system, the ease of user interface, automated notification, and the ability to update information including the attachment of files. Although direct comparison with the fault logbooks has not been made, anecdotally the consistency and utility of information provided on equipment faults has improved; however, the number of faults reported remains relatively stable. The system allows access by multiple users simultaneously from any computer in the cancer center, which is an improvement over a fault logbook which is physically located at the linac bunker. The automated email notification allows senior staff such as chief physicists and chief radiation therapists to be aware of evolving equipment issues impacting on clinical service provision in real time. The reporting feature allows assessment of service support performance over the long term, and is an evidence‐based tool for management of service contracts. The system has integrated well with the trend towards paperless departments, and field service reports (FSRs) are now received in electronic format and input into the system immediately.

As is the case for the traditional paper based logbook system, the data in SysAid is only as good as the quality of information provided. The system relies on users entering the correct categories and assigning an appropriate urgency. There are several instances in the ICCC database where an incorrect category has been entered into SysAid; typically this happens when multiple interlocks are present. For example, CTRL faults have been reported in SysAid as KFIL (klystron filament current below half the nominal value), as this is the first interlock that the linac software reports to the user. This can skew reporting trends and mask common problems. Another limitation of the system is that it is not possible to link multiple service requests that are related. If a fault occurs multiple times and has multiple entries, once the problem is resolved, each individual service request must be manually updated to reflect the status change. This limits the cleanliness of the database with multiple instances of faults that have not had their status updated. It is important that for the system to work well, a system administrator be assigned to manage the system. Compared to the time spent managing a traditional logbook, the new system requires far less time to extract the relevant fault information, but it does require one or two hours a month be spent by an administrator to maintain the database, add new users, and update categories.

The use of an online help desk system does not replace the need for communication between the radiation therapists, physicists, and engineers. There is always the potential that a problem entered into the system may never be discussed or actioned by the appropriate personnel because of the assumption that someone else is looking into it. Reporting the problem to a physicist in person, or over the phone, as well as entering the information into the system, is always preferable. The system does, however, have the advantage that it encourages reporting of more seemingly insignificant problems. For example, there is now a record of observations such as smells and noises in the linac bunker which previously may have been discussed but were never written down. The knowledge that every fault entered in SysAid automatically results in instant email notification to relevant staff encourages the recording of faults as soon as they occur. This is in contrast to the previous method of making an entry in a paper logbook where the perception was that the logbook would be infrequently read and therefore (if remembered), an entry could be made at a later time.

The examples that have been presented were focused on linear accelerator faults. However, the system at ICCC includes categories for the kilovoltage unit, the CT scanner, and the treatment planning system. There are also several features of the software that have not been implemented, such as asset management. In the near future the system will be expanded to incorporate fault reporting for the oncology information system and for the linear accelerators located at a new satellite site in Shoalhaven.

## V. CONCLUSIONS

The online help desk that has been implemented at the ICCC for equipment fault reporting has been found to be an effective alternative to the conventional paper‐based logbook. The software has allowed for equipment faults to be managed effectively and has added a level of traceability in fault management. The database features have enabled greater oversight of current and recurring machine problems, and have provided a means for ensuring that faults are followed up appropriately and that the effectiveness of the service performed is substantiated. The online help desk has not replaced the need for direct methods of verbal communication between the various professional groups involved with monitoring equipment performance; however, it has been a very useful tool for supporting that communication.

## ACKNOWLEDGMENTS

The authors would like to acknowledgement the contribution of the ICCC staff, in particular the radiation therapists and physicists, for providing the data upon which this manuscript is based.
